# A Case of Heat Exhaustion Masquerading as ST-Elevation Myocardial Infarction

**DOI:** 10.7759/cureus.30495

**Published:** 2022-10-19

**Authors:** Musab Egaimi, Heeyoung Seo, Sahla Bashir

**Affiliations:** 1 Cardiology Department, Sheikh Khalifa Specialty Hospital, Ras Al Khaimah, ARE

**Keywords:** stemi, myocardial infarction, st-elevation, rhabdomyolysis, heat stroke, heat exhaustion, heat-related illness

## Abstract

Heat-related illnesses (HRIs) are characterized by hyperthermia, neurological dysfunction, and multiorgan damage. Cardiac dysfunction has been reported, but ST-elevation with a rise in cardiac markers suggesting acute coronary syndrome has been infrequently reported. Data from the middle east in particular is lacking.

This is a case of a 43-year-old male patient from the United Arab Emirates diagnosed with acute inferior ST-elevation myocardial infarction necessitating cath lab activation revealing normal coronary arteries. Echocardiography did not show evidence of wall motion abnormalities. After reviewing his clinical presentation, he was diagnosed with heat exhaustion complicated by rhabdomyolysis and acute kidney injury. The patient fully recovered with intensive medical care.

This case showed that the electrocardiographic changes and elevation of cardiac markers do not reflect true cardiac ischemia but rather a pathophysiological response to HRI. Previously published reports were scarce and showed conflicting results due to the heterogenicity of cases and methods as well as the lack of angiographic documentation of coronary pathology.

This case report demonstrates the importance of early recognition and timely management of HRI cases with an unusual presentation that mimics myocardial infarction, especially in countries with high ambient temperatures during the summer seasons.

## Introduction

Heat-related illness (HRI) is a spectrum of disorders that exist in a continuum ranging from heat exhaustion to heat stroke and is characterized by thermodysregulation leading to extreme hyperthermia (can exceed 40.5°C), central nervous system dysfunction, and multiorgan failure [1,2]. HRI can occur either due to failure of heat dissipation during extreme environmental heat (classical type) or due to exertion when heat production exceeds the body’s ability to dissipate it (exertional type). The former usually occurs during heat waves, while the latter occurs sporadically and usually affects young active adults and athletes [1,3,4]. Heat stroke is the most severe form of HRI; serious complications usually arise with the onset of multiorgan-dysfunction syndrome, most notably, rhabdomyolysis and acute renal failure. Other notable complications include coagulopathy, acute respiratory distress syndrome (ARDS), hepatic dysfunction, and myocardial injury [1,5,6].

The cardiovascular system is frequently implicated in cases of HRI as it plays a key role in thermal regulation via the vascular convective heat transfer pathway. To meet tissue perfusion demands, the system is pushed to its limits, resulting in increased cardiac output, dilatation of cutaneous vessels, and constriction of splanchnic vessels so as to dissipate heat, combat hypovolemia, and decrease systemic vascular resistance produced by internal and external thermal loads [4,7]. Cardiac complications of heat exhaustion and heat stroke include electrocardiographic abnormalities (T-waves inversion, conduction abnormalities, arrhythmias, and transient ST-changes), regional wall motion abnormalities, congestive heart failure, cardiomyopathy, and cardiogenic shock [4].

Few cases of HRI with suspected ST-elevation myocardial infarction (STEMI) are recorded in the medical literature, and data from the middle east region is particularly scarce. This is a rare, documented case in the United Arab Emirates (UAE) of heat exhaustion presenting with ST-elevation compatible with the diagnosis of acute myocardial infarction (AMI) and subsequent cath lab activation.

## Case presentation

**Figure 1:**
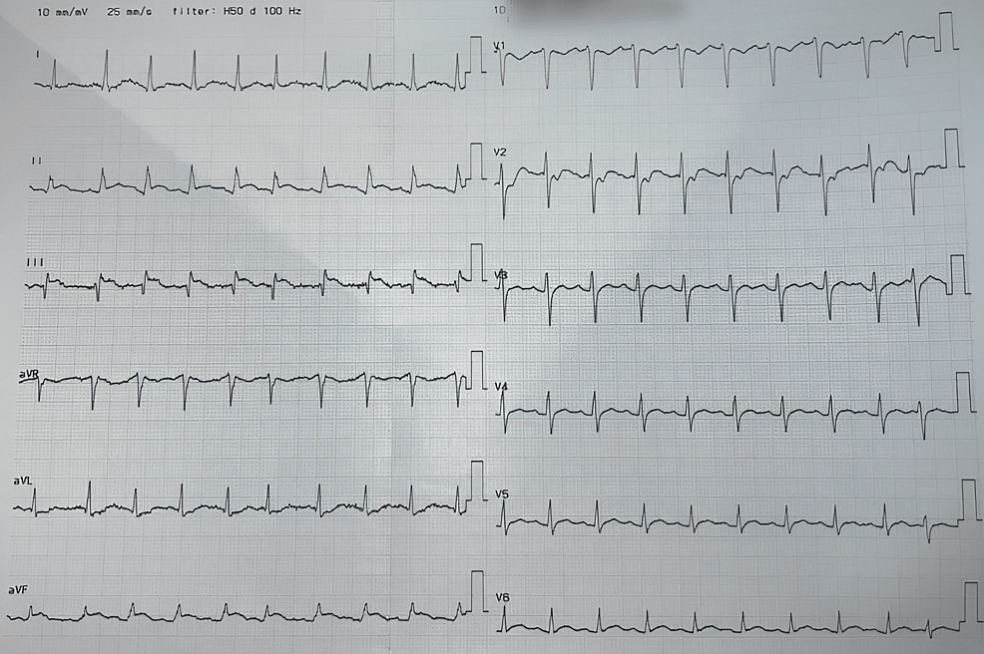
Initial ECG obtained via a secure, instant messaging communication channel from the referring hospital. The ECG shows ST-elevation > 1 mm in inferior leads suggesting a diagnosis of acute inferior STEMI ECG: Electrocardiogram; STEMI: ST-elevation myocardial infarction.

A 43-year-old male patient, with a known case of type 2 diabetes mellitus and hypertension, was referred from a nearby local hospital with a working diagnosis of inferior STEMI. He was initially brought in by the National Ambulance crew after he was found collapsed outdoors near his area of residence on a hot summer day with a recorded peak temperature of 42°C-43°C or (107.6°F-109.4°F). According to the paramedics, he was found in a confused state, dehydrated, ill, diaphoretic, and sweating profusely. He was complaining of shortness of breath and chest pain. He was immediately moved to a nearby community hospital, where he was evaluated and stabilized. An electrocardiogram (ECG) was obtained, which revealed ST-elevation of more than 1 mm in leads II, III, and aVF suggesting a diagnosis of inferior STEMI (Figure 1).

**Figure 2:**
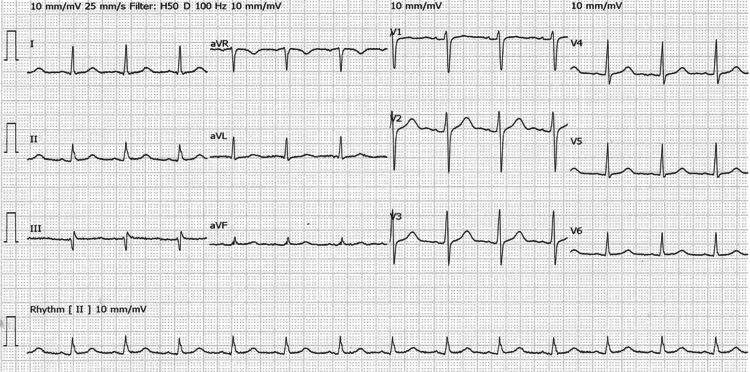
Subsequent ECG obtained immediately after diagnostic angiography showing complete resolution of ST-segment elevation in inferior leads ECG: Electrocardiogram; STEMI: ST-elevation myocardial infarction.

After a discussion with the interventional cardiologist, the patient was loaded with aspirin, ticagrelor, and heparin before being transferred to our facility. On arrival, the patient underwent coronary angiography (CAG) that showed insignificant coronary artery disease. Repeat ECG after CAG showed improved resolution of ST-segment changes (Figure 2). The patient recanted the history of chest pain on the cath lab table, claiming it was a just transient feeling of suffocation and breathlessness. The patient was then admitted to ICU for further evaluation and monitoring.

A bedside transthoracic echocardiography (TTE) did not show regional wall motion abnormality (RWMA), and his systolic function was normal with visually estimated left ventricular ejection fraction (LVEF) > 60%. A formal follow-up TTE confirmed these findings. The blood gas analysis was unremarkable (Table 1), and his laboratory results (Table 2) showed elevated cardiac markers (creatine kinase MB fraction [CK-MB] of 73.3 ng/mL and cardiac troponin T [cTnT] of 139 ng/L). His n-terminal pro b-type natriuretic peptide (NT-ProBNP) level was normal (23 pg/mL). Other remarkable laboratory results included leukocytosis (white blood cells count of 27.58 x 10³/μL), hyperbilirubinemia (total bilirubin of 1.9 mg/dL), and acute kidney injury (creatinine of 1.91 mg/dL and eGFR of 39 mL/min/1.73 m²), and his previous baseline was creatinine of 1.04 mg/dl and normal eGFR. After a careful review of the patient’s clinical narrative, a diagnosis of heat exhaustion followed by rhabdomyolysis was suspected. His creatine kinase level was elevated (23715 IU/L), as was his myoglobin level (>30000 ng/mL). Urine analysis showed significant albuminuria and myoglobinuria. The diagnosis of exhaustion followed by rhabdomyolysis and complicated acute kidney injury (AKI) was established, and the patient was started on aggressive fluid management in the ICU. The abdominal ultrasound did not show any significant kidney disease or other intra-abdominal pathology. His cTnT and CK-MB peaked 24 hours after admission at 789 ng/L and 203 ng/mL, respectively, while his NT-ProBNP peaked after 48 hours to reach 197 pg/mL. His CK-MB fell back to near-normal levels (14 ng/mL) after 72 hours of admission.

**Table 1:** Blood gas analysis results HCO_3_: Bicarbonate; O_2_ sat: Oxygen saturation; pCO_2_: Partial pressure of carbon dioxide; pH: Potential of hydrogen; pO_2_: Partial pressure of oxygen.

Parameters	Values	Reference Values	Unit
pH	7.40	7.38 ~ 7.46	
pCO_2_	31	32 ~ 46	mmHg
pO_2_	101	84 ~ 98	mmHg
HCO_3_^-^	20.6	21.0 ~ 29.0	mmol/L
Base excess	-5.7	-2 ~ 2	
O_2_ sat	98.8	92 ~ 96	%

**Table 2:** Key laboratory findings ALP: Alkaline phosphatase; ALT: Alanine transaminase; AST: Aspartate aminotransferase; BUN: Blood urea nitrogen; Ca: Calcium; CK-MB: Creatine kinase MB fraction; CRP: C-reactive protein; eGFR: Estimated glomerular filtration rate; K: Potassium; Na: Sodium; NT-ProBNP: n-terminal pro b-type natriuretic peptide; WBC: White blood tests.

Parameters	Initial Values	Peak Values	Discharge Values	Reference Values	Unit
Creatine kinase	23715	23715	288	39 ~ 308	IU/L
Myoglobin	>30000	>30000	158	28 ~ 72	ng/mL
CK-MB	73.3	203	14	0 ~ 6.22	ng/mL
Troponin T	139	789	527	0 ~ 14	ng/L
#CRP	2.41	9.68	1.15	0.00 ~ 0.50	mg/dl
#NT-ProBNP	23	193	32	0 ~ 125	pg/mL
WBC	27.58	27.58	8.69	4.00 ~ 10.00	x10³/uL
Na	138	139	137	136 ~ 145	mmol/L
K	3.6	4.6	4.0	3.5 ~ 5.1	mmol/L
Ca	8.2	9.3	8.8	8.6 ~ 10	mg/dL
BUN	20	22	15	6 ~ 20	mg/dL
Creatinine	1.59	1.91	1.11	0.70 ~ 1.20	mg/dL
eGFR	48	39	72	60~	mL/min/1.73m²
Total bilirubin	1.9	1.9	0.8	0 ~ 1.2	mg/dL
ALP	143	156	163	40 ~ 129	IU/L
AST	169	489	38	0 ~ 40	IU/L
ALT	63	111	45	0 ~ 41	IU/L

Management continued under a multidisciplinary team with continuous hydration and careful monitoring of his electrolyte balance and urine output. His renal function gradually improved to near baseline levels at discharge (creatinine decreased to 1.11 mg/dl and eGFR improved to 72 mL/min/1.73 m²). His other key parameters (cTnT, creatine kinase, and myoglobin) improved as well. On day seven, he was discharged in fair condition with follow-up instructions.

## Discussion

Data on the prevalence of ST-elevation with or without ischemia among the HRI population is scarce. Most of the data is derived from small cohort studies and sporadic case reports. According to data from the Arabian peninsula, ST-elevation occurs in nearly 50% of heat stroke cases, and the ischemic picture of ST-elevation, RWMA, and elevation in cardiac enzymes occur in approximately 25% of cases [4,8]. Data from a cohort study in the United States found that AMI occurs in 7% of heat stroke cases, with only 8% receiving coronary angiography and 0.5% receiving interventional treatment [9]. A report on a series of cases during the Australian heat wave of 2013 showed that two out of four patients had ST-elevation that resolved spontaneously after cooling but without echocardiographic abnormalities [10]. Diabetes, hypertension, and obesity as with this patient are believed to be risk factors for myocardial infarction in HRI cases, but the evidence supporting the claim is limited [9].

Angiographic evaluation of HRI patients with ST-elevation or increased cardiac markers is rarely reported in the literature. Rajan et al. reported a case in Kuwait of a 23-year-old man from Bangladesh who presented with presumed anterolateral STEMI but a normal coronary angiogram [11]. Chen et al. reported a similar case of a 39-year-old man in Kuwait with HRI who was initially diagnosed with anterolateral STEMI before it was revealed that his coronaries were patent [12]. Both cases, however, were later diagnosed with stress-induced cardiomyopathy secondary to HRI.

Our patient showed dynamic rise and fall of cardiac markers usually seen in myocardial injury but without documented RWMA or angiographic findings. High levels of serum cardiac markers in cases of HRI have been reported, but neither their predictive value nor their prognostic value is established due to the paucity of reported data [13,14]. A post-doc analysis of 514 patients showed that cardiac troponin I (cTnI) was elevated in more than 50% of subjects, and severe myocardial damage was detected in less than 20% [13]. One of the major drawbacks of this study is that it inferred myocardial damage through high cTnI levels (cutoff point of >1.5 ng/mL^1^) rather than electrocardiographic, echocardiographic, or angiographic evidence.

Similarly, a case report of heat stroke showed elevation in cTnI in a 21-year-old male with exertional HRI that normalized within eight days but without documenting myocardial damage [14]. Elevated cardiac troponin T (cTnT) levels were also reported in up to 60% of exertional HRI without documentation of myocardial injury [15]. Cardiac markers are known to be elevated as a physiological response to strength training or pathological condition such as critical illness, sepsis, and renal impairment [16]. Thus, it is likely that the rise of cardiac troponins during HRI reflects the state of the underlying condition rather than being a marker of true myocardial injury. The mechanisms behind the elevated levels of troponin are not clear; some of the postulated mechanisms suggest either reduced elimination with falling glomerular filtration or enhanced release secondary to mechanical stress, cell membrane leakage, cytotoxic damage, or neurally mediated cell necrosis [13,14,16-18].

Late cardiac complications such as ischemic heart disease, heart failure, and atrial fibrillation were reported in cases of HRI and are thought to occur due to delayed metabolic disturbances and the enhancement of atherosclerosis [19].

## Conclusions

This case highlights the unusual presentation of HRI, which can mimic STEMI. Proper clinical evaluation and sound clinical judgment are key to avoiding unnecessary invasive evaluation in this group of patients, especially in countries with high ambient temperatures in the summer seasons. Given the rarity of these cases being reported in the medical literature, it is debatable whether the changes in the ECG and cardiac markers indicate genuine cardiac ischemia.
